# Membrane cholesterol regulates TRPV4 function, cytoskeletal expression, and the cellular response to tension

**DOI:** 10.1016/j.jlr.2021.100145

**Published:** 2021-10-25

**Authors:** Monika Lakk, Grace F. Hoffmann, Aruna Gorusupudi, Eric Enyong, Amy Lin, Paul S. Bernstein, Trine Toft-Bertelsen, Nanna MacAulay, Michael H. Elliott, David Križaj

**Affiliations:** 1 Department of Ophthalmology & Visual Sciences, University of Utah School of Medicine, Salt Lake City, UT, USA; 2 Dean A. McGee Eye Institute, University of Oklahoma Health Sciences Center, Oklahoma City, OK, USA; 3 Department of Neuroscience, University of Copenhagen, Copenhagen, Denmark; 4 Department of Bioengineering, University of Utah, Salt Lake City, UT, USA; 5 Department of Neurobiology, University of Utah, Salt Lake City, UT, USA

**Keywords:** cell signaling, cyclodextrins, dyslipidemias, eye/retina, glaucoma, lipid rafts, mechanotransduction, smooth muscle cells, TRPV4, αSMA, α-smooth muscle actin, [Ca^2+^]_i_, intracellular calcium concentration, C/PC, cholesterol/phosphatidylcholine, Cav-1, caveolin-1, ECM, extracellular matrix, F-actin, filamentous actin, HTS, hypotonic stimuli, IOP, intraocular pressure, ir, immunoreactivity, MβCD, m-β-cyclodextrin, NA, numerical aperture, PBS, phosphate-buffered saline, ROI, region of interest, TM, trabecular meshwork, TMCM, trabecular meshwork cell medium, TRPV4, transient receptor potential vanilloid isoform 4

## Abstract

Despite the association of cholesterol with debilitating pressure-related diseases such as glaucoma, heart disease, and diabetes, its role in mechanotransduction is not well understood. We investigated the relationship between mechanical strain, free membrane cholesterol, actin cytoskeleton, and the stretch-activated transient receptor potential vanilloid isoform 4 (TRPV4) channel in human trabecular meshwork (TM) cells. Physiological levels of cyclic stretch resulted in time-dependent decreases in membrane cholesterol/phosphatidylcholine ratio and upregulation of stress fibers. Depleting free membrane cholesterol with m-β-cyclodextrin (MβCD) augmented TRPV4 activation by the agonist GSK1016790A, swelling and strain, with the effects reversed by cholesterol supplementation. MβCD increased membrane expression of TRPV4, caveolin-1, and flotillin. TRPV4 did not colocalize or interact with caveolae or lipid rafts, apart from a truncated ∼75 kDa variant partially precipitated by a caveolin-1 antibody. MβCD induced currents in TRPV4-expressing *Xenopus laevis* oocytes. Thus, membrane cholesterol regulates trabecular transduction of mechanical information, with TRPV4 channels mainly located outside the cholesterol-enriched membrane domains. Moreover, the biomechanical milieu itself shapes the lipid content of TM membranes. Diet, cholesterol metabolism, and mechanical stress might modulate the conventional outflow pathway and intraocular pressure in glaucoma and diabetes in part by modulating TM mechanosensing.

## Graphical abstract

**Figure:**
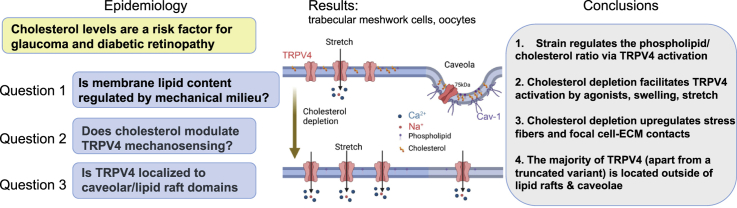

Conversion of sensory information into electrical and chemical signals in eukaryotic cells is modulated by unesterified cholesterol, a planar 27-carbon polycyclic molecule that constitutes ∼20% of the total mass of membrane lipids (1, 2). Its intercalation into phospholipids reduces the motion of hydrocarbon chains, affects surface charge, and promotes membrane stiffness while keeping the membrane fluid (3, 4, 5). Cholesterol is a precursor for steroid hormones and regulates stereospecific interactions with transmembrane channels, transporters, and enzymes within membrane-bound glycosphingolipid-rich protein complexes (lipid rafts; (6)) that serve as organizing hubs for intracellular signaling pathways, membrane trafficking, cytoskeleton, and cell-extracellular matrix (ECM) interactions (1, 2, 7, 8). Alterations in free membrane cholesterol and lipid raft density positively and negatively affect membrane channels, with little consensus about the molecular mechanisms (9, 10, 11).

Vertebrates maintain cholesterol levels within a narrow range, with deficits or oversupply often harmful for health (5, 6). Defects in cholesterol regulation contribute to atherosclerosis, myocardial injury, diabetic and vascular dysfunctions (12, 13, 14), and increase the risk for progression of neurodegenerative diseases such as primary open-angle glaucoma, a prevalent cause of blindness (15, 16). Patients with high serum cholesterol levels have an increased likelihood for elevated intraocular pressure (IOP), with glaucoma linked to multiple genes involved in cholesterol metabolism (17, 18). A principal regulator of IOP is the trabecular meshwork (TM), a multilayered tissue composed of smooth muscle-like cells with mechanosensitive and contractile functions (16, 19, 20, 21). In response to mechanical stress and glaucoma, TM cells upregulate actomyosin cytoskeleton, ECM secretion, and the size/number of focal cell-ECM contacts, thereby increasing cell contractility and tissue resistance to the outflow of aqueous humor (22, 23, 26, 27). It is not known how TM membrane lipid composition is affected by the biomechanical milieu and whether changes in membrane stiffness impelled by free membrane cholesterol affect TM mechanosensing, intracellular signaling, and cytoskeletal organization.

We recently identified the mechanosensitive transient receptor potential vanilloid 4 (TRPV4) channel as a principal regulator of mechanically induced signaling in mouse and human TM (20, 26, 27). This ubiquitous nonselective cation channel transduces the effects of membrane strain, shear flow, swelling, and thermal stimuli into calcium signals, which control a wide range of downstream signaling pathways (21, 26, 28, 29). The TRPV4 sequence appears to have coevolved with enzymes linked to cholesterol biosynthesis pathways (30). Studies in endothelial cells suggested that TRPV4 is trafficked to cholesterol- and caveolin-1 (Cav-1)-enriched rafts (31) to modulate vascular flow (32) but the interactions between mechanotransduction, TRPV4 signaling, and cholesterol are not well understood. We used human TM cells and an oocyte overexpression system to investigate cholesterol-dependent modulation of TRPV4 signaling in model mammalian and nonmammalian systems. Interestingly, phosphatidylcholine (PC) versus cholesterol content of TM membranes was regulated by stretch in a TRPV4-dependent manner. TRPV4 was mainly located in nonraft regions, as indicated by the lack of interaction and absence of colocalization with caveolar and noncaveolar components of lipid rafts. Lowering the levels of free membrane cholesterol facilitated TRPV4 activation and promoted cytoskeletal polymerization. These findings suggest a potential mechanism whereby diet, the biomechanical milieu, and systemic/local cholesterol homeostatically regulate the aqueous outflow pathway.

## Materials and methods

### TM cell culture and isolation

Primary cultures of TM cells were dissected from three eyes of donors with no history of eye disease (65-year-old male, 68-year-old male, and 78-year-old male) as described previously (26, 27, 33) and in concordance with the tenets of the World Medical Association Declaration of Helsinki and the Department of Health and Human Services Belmont Report. A subset of experiments was conducted in immortalized juxtacanalicular human TM cells obtained from ScienCell (catalog no. 6590) and used up to the seventh passage. Primary and immortalized human TM cells showed no differences in responses to TRPV4 or cholesterol-modulating agents. Cells were grown in trabecular meshwork cell medium (TMCM; ScienCell; catalog no. 6591) at 37°C and 5% CO_2_. The phenotype was periodically validated by profiling for markers, including *Aqp1*, *Timp3*, *Myoc*, *MGP*, *Acta2* (α-smooth muscle actin [αSMA]), and dexamethasone-induced upregulation of myocilin expression. These data are shown in our previous characterizations of the cell line (21, 26, 33).

*Xenopus laevis* oocyte experiments were performed according to the guidelines of the Danish Veterinary and Food Administration (Ministry of Environment and Food) and approved by the animal facility at the Faculty of Health and Medical Sciences, University of Copenhagen. The experiments conform to the principles and regulations described (34). The surgical protocol by which the oocytes were retrieved was approved by The Danish National Committee for Animal Studies, Danish Veterinary and Food Administration (Ministry of Environment and Food). This work appeared previously in abstract and preprint forms (35, 36).

### Reagents

The TRPV4 agonist GSK1016790A (GSK101) and cholesterol were obtained from Sigma or VWR. GSK101 (1 mM) stock aliquots were prepared in dimethyl sulfoxide and subsequently diluted into working saline concentrations (5 and 25 nM, respectively). Chemical reagents for biochemical experiments—methanol, isopropanol, *n*-hexane, and chloroform—were of GC/MS grade and purchased from Thermo Fisher Scientific. The cholesterol standard was purchased from Sigma-Aldrich.

### Cholesterol depletion and repletion

Methyl-β-cyclodextrin (MβCD; Sigma; C4555) was dissolved in TMCM and used at 10 mM, the concentration that removes 80–90% of free membrane cholesterol (37, 38). Cells were preincubated with MβCD for 60 min to maximize extraction, washed in TMCM, and placed into recording chambers for optical recordings. This protocol maintains decreased membrane cholesterol levels for at least 24 h (39). Supplementation was based on perfusion with cholesterol-saturated MβCD. Powdered cholesterol was dissolved for 30 min in 80% ethanol solution at 75–80°C to obtain a 10 mM stock solution. The stock was dissolved in TMCM containing MβCD to the final cholesterol concentration of 1 mM. A parallel chamber used the cholesterol stock to load MβCD for the final concentrations of 1 mM cholesterol + 10 mM MβCD.

### Hypotonic stimulation

The swelling studies were conducted as reported previously (40, 41). Extracellular NaCl was kept at 57.5 mM, and total osmolarity was regulated by adding or removing mannitol, a procedure that maintains the ionic strength of the extracellular solution. Osmolarity was checked thermometrically using a vapor pressure osmometer (Wescor).

### Lipid extraction and GC/MS chromatography

Lipids were extracted using Folch method (42). Total lipids were extracted by adding methanol/chloroform/water (1:2:1, v/v). The chloroform phase was washed to remove water residues and dried under nitrogen gas. The dried film was dissolved in 100 μl hexane, and 5 μl of the sample was injected into the GC/MS instrument for cholesterol analysis. The Thermo Trace GC-DSQ II system (Thermo Fisher Scientific) consists of an automatic sample injector (AS 3000), gas chromatograph, single quadrupole mass detector, and an analytical workstation. Chromatographic separation was carried out with an Rxi-5MS-coated 5% diphenyl/95% dimethyl polysiloxane capillary column (30 m × 0.25 mm inner diameter, 0.25 μm film thickness) (Restek Corporation, PA). The sample was injected into the GC/MS using a splitless mode, the septum purge was on, and the injector temperature was set at 200°C. The column temperature was programmed as follows: initial temperature 60°C, 15°/min to 240°C, 2°/min to 290°C, and a hold at 290°C for 5 min. Transfer line temperature was 290°C. Helium was used as the carrier gas at a flow rate of 1.5 ml/min. MS conditions were as follows: electron ionization mode with ion source temperature of 250°C and multiplier voltage, 1,182 V; full scan and selected ion monitoring mode was used to identify and quantify cholesterol. The area values of cholesterol were plotted against known range of standards (100–0.1 ng) to quantify the cholesterol in the samples. PC levels were measured by a colorimetric/fluorometric assay kit (BioVision).

### Immunoprecipitation and Western blot analysis

Cell lysis was performed in lysis buffer containing 2% octylglucoside, 150 mM NaCl, 10 mM Tris-HCl, pH 7.4, 0.5 mM EDTA, 0.1% Triton X-100, and protease inhibitor cocktail (Roche). Lysates were cleared by centrifugation, and protein concentrations determined using a BCA assay (Thermo Fisher Scientific). Immunoprecipitation of Cav-1 was performed using Dynabeads™ Protein G Kit (Thermo Fisher Scientific) according to the manufacturer's instructions. Briefly, Cav-1 primary antibody (10 ng/μl) was conjugated to Dynabeads magnetic beads at room temperature. Then, an equal amount of protein from each sample was incubated with the beads-antibody conjugates for 15 min at room temperature, with gentle agitation. The beads were removed from solution by DynaMag™-2 magnet (Thermo Fisher Scientific), washed in wash buffer, and resuspended in lysis buffer. The original cell lysates, immunoprecipitates, and unbound fractions (flow-through) were boiled in Laemmli buffer, separated by reducing SDS-PAGE and transferred to nitrocellulose membranes. Membranes were blocked with 5% BSA for 1 h, and probed with Cav-1 (Cell Signaling Technology) and TRPV4 primary antibodies (Alomone Lab). Primary antibodies were detected using Clean-Blot® IP detection reagent (Thermo Fisher Scientific) conjugated to HRP.

### Detergent-free lipid raft isolation and Western blot analysis

Samples were homogenized in hypotonic homogenization buffer (20 mM Tris-HCl, pH 7.8, 3 mM MgCl_2_, 10 mM NaCl, 0.0005 mg/ml, 2 mM sodium vanadate, 20 mM sodium fluoride, 0.5 mM DTT, and 1 mM PMSF) on ice and centrifuged at 15,000 *g* for 30 min at 4°C to separate cytosolic proteins from intracellular and plasma membranes. The pellet was resuspended in 0.5 M Na_2_CO_3_, transferred to a 5%/35%/45% sucrose (in Na_2_CO_3_) flotation gradient and spun at 36,000 rpm for 18 h using a preparative ultracentrifuge model XL-90 (NVT90 rotor; Beckman Coulter Life Sciences). Fractions obtained from the sucrose gradient were diluted in hypotonic buffer and spun at 15,000 *g* for 30 min at 4°C. Pellets (25 μl) were resuspended in RIPA buffer and 2× Laemmli buffer. About 30 μl of each sample was loaded in 10% SDS-PAGE and transferred to PVDF membranes for 1 h at 220 mA. Nonspecific binding was blocked with 5% nonfat milk and 2% BSA. The samples were incubated overnight at 4°C with TRPV4 (1:500; Alomone Labs), flotillin (1:200; Santa Cruz Biotechnology), Cav-1 (1:1,000; Cell Signaling), and α-SMA (1:500; Sigma-Aldrich) antibodies, followed by anti-mouse (1:5,000; BioRad) or anti-rabbit (1:5,000; Cell Signaling) HRP-conjugated secondary antibodies. The blotted proteins were developed with an enhanced chemiluminescence kit (Thermo Fisher Scientific).

### Immunofluorescence

Cells were fixed with 4% paraformaldehyde for 10 min. After a phosphate-buffered saline (PBS) rinse, PBS containing 5% FBS and 0.3% Triton X-100 blocking solution was applied for 20 min. Filamentous actin (F-actin) was labeled with AlexaFluor 488 phalloidin (1:1,000; Life Technologies). Primary antibodies (rabbit anti-TRPV4, 1:1,000, Lifespan Biosciences; mouse antiflotillin, 1:200, Santa Cruz; and mouse anticaveolin, 1:1,000, BD Biosciences) were diluted in antibody solution (2% BSA and 0.2% Triton X-100 in PBS) and applied overnight at 4^°^C. The TRPV4 antibody does not label TRPV4 KO tissues (43, 44). After rinsing, slices were incubated with secondary antibodies diluted to 1:1,000 in PBS for 1 h at room temperature. Plasma membrane cholesterol was tracked with filipin (Sigma; F9765). As previously described (45), 0.005% filipin (Sigma) was dissolved in dimethyl sulfoxide and applied to dissociated cells together with the secondary antibody (goat anti-rabbit AlexaFluor 488; 1:500; Life Technologies). Unbound antibodies were rinsed, and conjugated fluorophores were protected with Fluoromount-G (Southern Biotech) prior to coverslipping. Images (10 per experiment) were acquired on Olympus CV1200 confocal microscope using a NeoFluor 20× water immersion objective.

### Analysis and particle counting

Images were acquired using identical parameters (HV, gain, and offset), resulting in very similar signal-to-noise ratios across datasets. ImageJ (National Institutes of Health) was used to extract and quantify the mean intensities and particle analysis of immunoreactive signals, with ∼40–50 cells per slide averaged across at least three independent experiments. The fluorescence intensity of F-actin was measured in arbitrary units using the area integrated intensity measurement tool of ImageJ with background compensation. Data were plotted as the signal as averaged and normalized fluorescence intensity (in percent) per cell area compared with the control. In particle analysis, color images were converted to black and white using *Binary → Convert to mask* with white background and automatic threshold level. Immunoreactive puncta (number/cell area) with the segmented area were counted with the *Analyze particles* plug-in. Minimum (3 pixel^2^) and maximum (30 pixel^2^) pixel area sizes were defined to exclude regions outside the regions of interest (ROIs), calculate the particle number/cell area, and determine the relative puncta numbers. Individual particle sizes for each cell were averaged and normalized.

### Optical imaging

Calcium responses in TM cells were tracked following published protocols (43, 46, 47). Briefly, for charge-coupled device imaging, cells were loaded with 5–10 μM Fura-2-AM for 45 min and perfused with isotonic saline (pH 7.4) containing (in millimolar): NaCl 133, KCl 2.5, NaH_2_PO_4_ 1.5, MgCl_2_ (6H_2_0) 1.5, CaCl_2_ 2, glucose 10, Hepes hemisodium salt 10, pyruvic acid 1, lactic acid 1, l-glutamine 0.5, glutathione 0.5, sodium ascorbate 0.3, with pH 7.4, and osmolarity at 300 ± 10 mOsm, delivered through a gravity-fed 8-reservoir system (Warner Instruments) that converged toward a manifold tube inserted into the experimental chamber. Epifluorescence was detected with Photometrics Delta or Prime BSI cameras. Ratiometric Ca^2+^ imaging was performed on ROIs that marked a central somatic region and were typically binned at 3 × 3 (43, 46). Background fluorescence was measured in similarly sized TM ROIs in neighboring areas devoid of cells. The microscopes were inverted with a Nikon Ti with 40× (0.75 numerical aperture [NA] oil) or upright Nikon E600 FN microscopes with a 20× (0.8 NA water) and 40× (1.3 NA oil and 0.8 NA water) objectives. A wide-spectrum 150 W Xenon arc lamp (DG4; Sutter Instruments, Novato, CA) provided excitation to 340 and 380 nm filters (Semrock). The signals were analyzed using NIS-Elements Advanced Research 3.2 and MS. Excel Δ*R*/*R* (peak *F*^340^/*F*^380^ ratio − baseline/baseline) was used to quantify the amplitude of Ca^2+^ signals. Data acquisition and F340/F380 ratio calculations were performed by NIS Elements 3.22 software (Melville, NY).

### Spectrophotometry

The intracellular calcium concentration [Ca^2+^]_i_ was monitored using a plate reader (Turner Biosystems). Cells were seeded onto noncoated 96-well plates and loaded with 2 μM of Fluo-4 AM for 30–45 min at 37°C. Fluorescence was measured at an excitation of 490 nm and an emission of 520 nm, with intervals of 90 s and 6–10 measurements per experiment. About 490/520 nm ratios were normalized to the control untreated samples. Baseline measurements were recorded in control wells at the same time without addition of agonist or hypotonic stimuli (HTS).

### Membrane strain assay

**Fig. 1:**
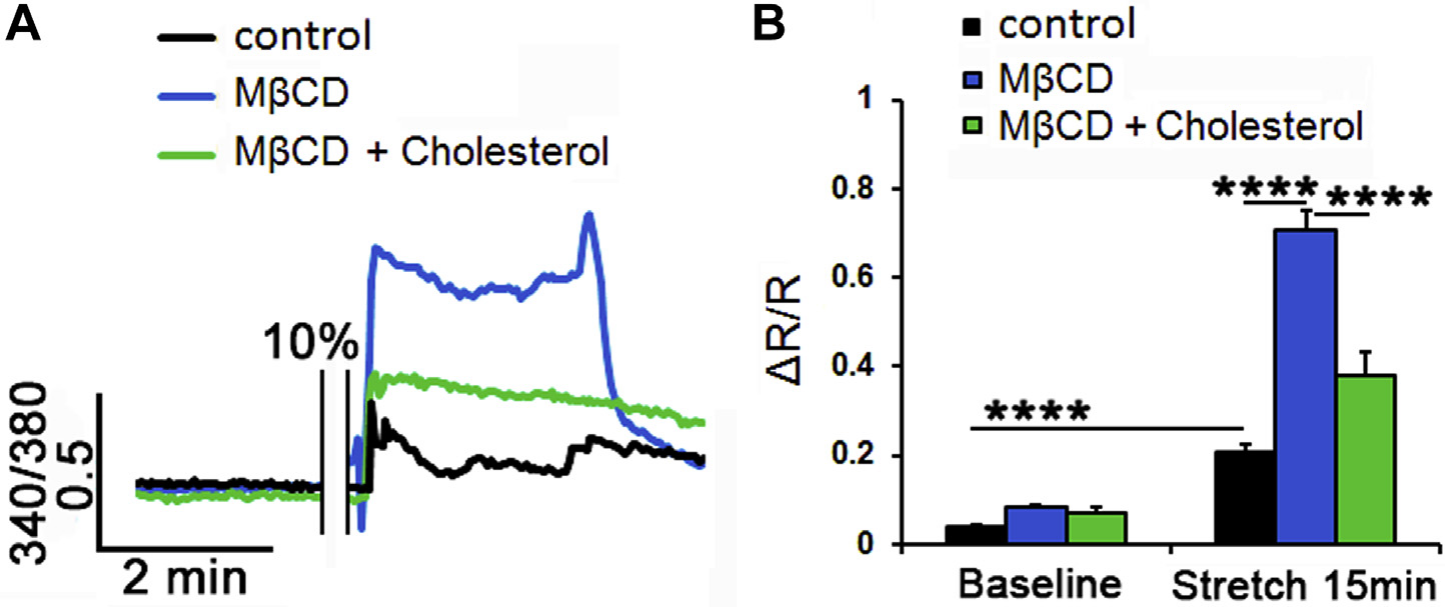
Membrane cholesterol modulates the cyclic stretch-induced intracellular calcium responses in TM cells. A: Representative traces. B: Averaged results for 15 min cyclic stretch of control (black trace, bars), MβCD-treated (10 mM, blue trace, bar), and MβCD:cholesterol-treated (green trace, bar) TM cells. Stretch-evoked [Ca^2+^]i responses are modulated by membrane cholesterol (n = 21–77; N = 5–6). ∗∗∗∗*P* < 0.0001.

TM cells were seeded on flexible silicon membranes coated with type I/IV collagen, grown to 80% confluence, and placed into a FlexJunior chamber controlled by the Flexcell-5000 Tension system (Flexcell) (26). Stretch-induced Ca^2+^ influx and cytoskeletal changes were tracked in cells loaded with Fura-2-AM for 30–60 min and stimulated with cyclic biaxial stretch (10%, 1 Hz, 15 min, or 6%, 0.5 Hz 1–3 h, respectively) at 37°C. Changes in focal plane (which disrupted calcium imaging for several seconds, as indicated by breaks in response trace in Fig. 1A) were adjusted manually. Cells were imaged with a Nikon E600FN upright microscope. Excitation light was provided by a Xenon source within a Lambda DG4 (Sutter Instruments) and controlled by Nikon Elements.

**Fig. 5:**
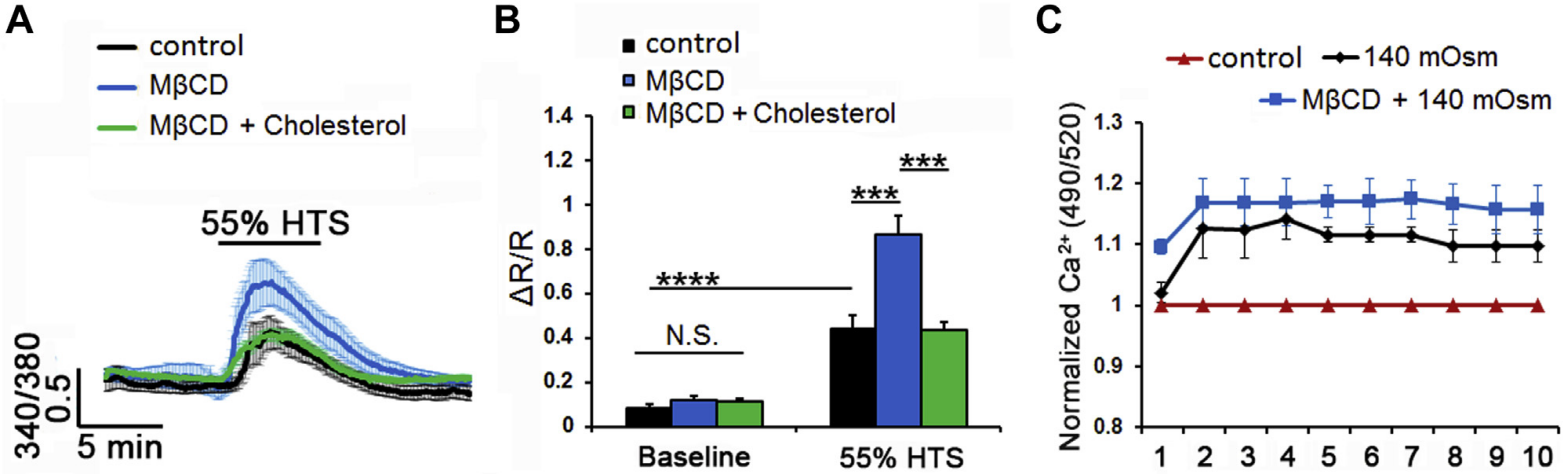
Cholesterol depletion facilitates HTS-induced Ca^2+^ signals. A, B: Cytosolic Ca^2+^ responses in Fura-2 AM-loaded cells. A: HTS induced [Ca^2+^]_i_ elevations in representative control, MβCD and MβCD:cholesterol-treated samples (n = 8–10). B: Averaged data from A. MβCD (blue bar) augmented, whereas MβCD:cholesterol (green bar) reduced HTS-induced [Ca^2+^]_i_ increases. C: Fluorimetry. 55% HTS (140 mOsm) increased Fluo-4 signals. This effect was facilitated by MβCD (N = 3). ∗∗∗*P* < 0.001; ∗∗∗∗*P* < 0.0001; NS, nonsignificant.

**Fig. 2:**
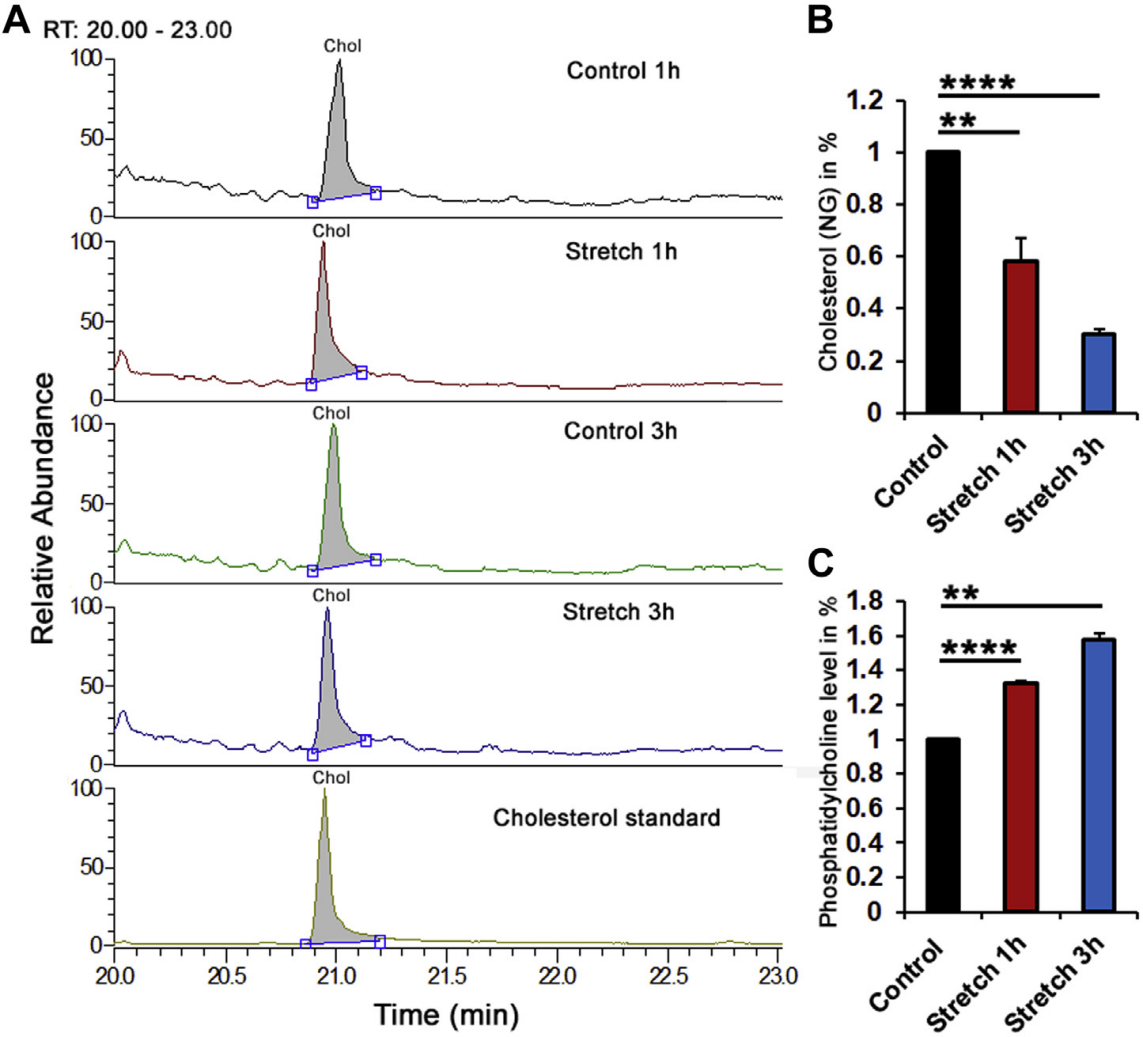
The C/P ratio in TM membranes is stretch dependent. A: Representative chromatograms. B: Normalized and averaged cholesterol GS/MS data in control, 1 and 3 h stretched primary TM cells. C: Normalized and averaged PC fluorometric data in control, 1 and 3 h stretched primary TM cells. Stretch induced a time-dependent decrease in membrane cholesterol level, which was concomitant with increasing content of PC membrane. N = 3; ∗∗*P* < 0.01; ∗∗∗∗*P* < 0.0001.

**Fig. 3:**
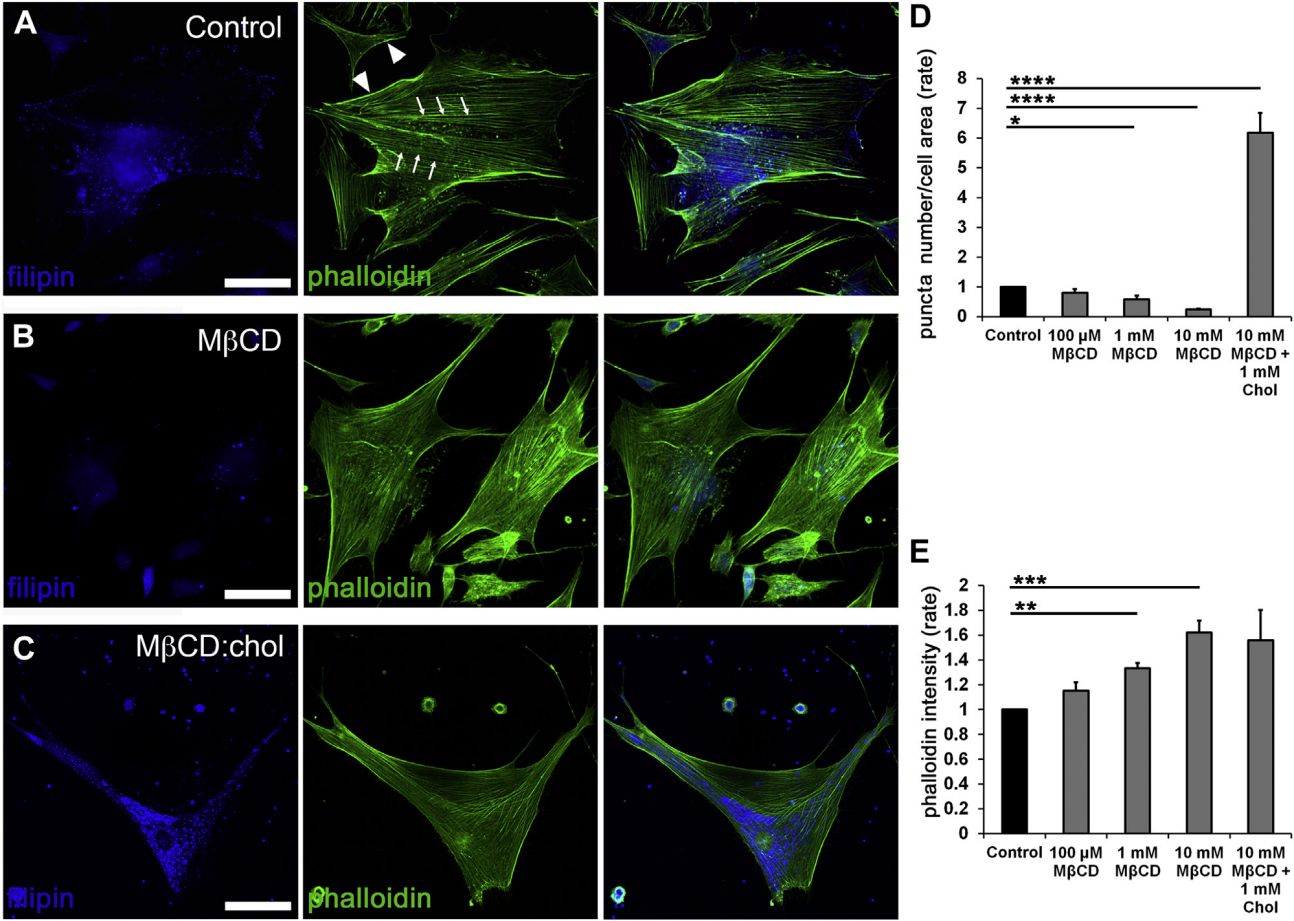
Cholesterol depletion promotes formation of actin stress fibers. Double labeling for F-actin (phalloidin-Alexa 488 nm) and filipin (405 nm). A: Untreated preparations show typical stress fiber organization dotted by lipid rafts. B: About 1 h of incubation with MβCD results in dissolution of filipin^+^ puncta and upregulation of phalloidin-actin fluorescence. C: Saturated 1:10 admixture of cholesterol (1 mM) and MβCD (10 mM) increased the number of filipin puncta. D, E: Averaged data for experiments shown in A–C (N = 3–4). ∗*P* < 0.05, ∗∗*P* < 0.01, ∗∗∗*P* < 0.001, and ∗∗∗∗*P* < 0.0001. The scale bar represents 20 μm.

**Fig. 4:**
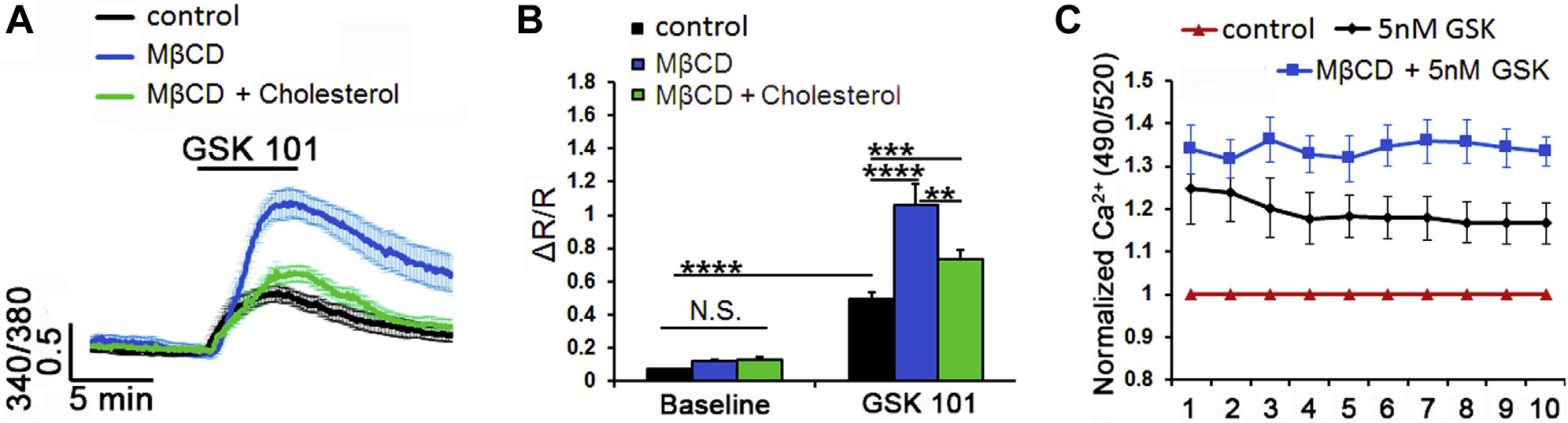
Cholesterol depletion increases the amplitude of TRPV4 agonist-induced Ca^2+^ signals. A, B: Ratiometric signals in Fura-2 AM-loaded cells. A: GSK101-induced elevations are increased in MβCD-treated cells (n = 8–10). B: Averaged data. MβCD (blue bar) augmented, whereas MβCD:cholesterol (green bar) reduced the amplitude of agonist-induced fluorescence. C: Fluorimetry, cell populations in 96 wells. About 5 nM GSK101 increased Fluo-4 fluorescence. Its effect was facilitated (∼12%) by MβCD (N = 4). ∗∗*P* < 0.01; ∗∗∗*P* < 0.001; ∗∗∗∗*P* < 0.0001. NS, nonsignificant.

### RNA preparation and heterologous expression in *Xenopus laevis* oocytes

*Xenopus laevis* frogs were obtained from Nasco (Fort Atkinson). The frogs were kept in tanks in a recirculating water facility and fed twice weekly with Floating Frog Food 3/32 for adults and juveniles (Xenopus Express, Inc). Oocytes were surgically removed from anesthetized frogs (41, 48) under anesthesia (2 g/l tricain, 3-aminobenzoic acid ethyl ester; Sigma A-5040). Preparation of defolliculated oocytes was carried out as described (49). Oocytes were kept in Kulori medium (in millimolar): 90 NaCl, 1 KCl, 1 CaCl_2_, 1 MgCl_2_, 5 Hepes (pH 7.4). Complementary DNA encoding rat TRPV4 was subcloned into the oocyte expression vector pXOOM, linearized downstream from the poly-A segment, and in vitro transcribed using T7 mMessage machine according to the manufacturer's instructions (Ambion). Complementary RNA was extracted with MEGAclear (Ambion) and microinjected into defolliculated *Xenopus laevis* oocytes: 4 ng TRPV4 RNA/oocyte. A TRP channel antagonist, ruthenium red (1 μM; Sigma-Aldrich; R-2751), was added to the medium to prevent tonic TRPV4-mediated currents mediating oocyte lysis. The oocytes were kept for 3–4 days at 19°C before experiments.

### Electrophysiology on *Xenopus laevis* oocytes

Conventional two-electrode voltage-clamp studies were performed with a DAGAN CA-1B High-Performance oocyte clamp (DAGAN) with Digidata 1440A interface controlled by pCLAMP software, version 10.5 (Molecular Devices). Electrodes were pulled (HEKA and PIP5) from borosilicate glass capillaries to a resistance of 2.5–3 MΩ when filled with 1 M KCl. The current traces were obtained in a test solution containing (in millimolar): 50 NaCl, 2 KCl, 1 MgCl_2_, 1 CaCl_2_, 10 Hepes, 100 mM mannitol (Tris-buffered pH 7.4, 220 mOsm) by stepping the clamp potential from −20 mV to test potentials ranging from +50 to −130 mV (pulses of 200 ms) in increments of 15 mV. Recordings were low pass filtered at 500 Hz, sampled at 1 kHz, and steady-state current activity analyzed at 140–180 ms after applying the test pulse. Depletion of endogenous cholesterol in intact oocytes was induced with 50 μM MβCD for 45 min (e.g., 55). All experiments were performed at room temperature (23°C).

### Data analysis

Statistical analyses were performed with GraphPad Prism 6.0 (GraphPad) and Origin Pro 8.5 (OriginLab). Data were acquired from at least three different experimental preparations on different days, with 3–6 slides/experiment. Typically three different batches of oocytes were used. Unless indicated otherwise, unpaired or paired *t*-tests were used to compare two means, and a one-way ANOVA along with the Tukey test was used to compare three or more means. Means are shown ± SEM. *P* > 0.05 = not significant; *P* < 0.05 = ∗, *P* < 0.01 = ∗∗, *P* < 0.001 = ∗∗∗, and *P* < 0.0001 = ∗∗∗∗. Preliminary versions of this study were published in abstract (35) and preprint (36) forms.

## Results

### The cholesterol/phosphatidylcholine ratio of the TM membrane is regulated by tensile stretch

The mechanical properties of the lipid bilayer can change under tension (50), but it is not known whether the biomechanical milieu shapes the membrane lipid content. To test this, we assessed changes in cholesterol content and the C/PC (cholesterol/PC) molar ratio following the stimulation of cells with cyclic mechanical strain. Primary human TM cells were isolated from healthy donors (26, 27), plated on ECM (collagen I)-coated membranes, and stimulated for 1 or 3 h with radial stretch (0.5 Hz; 6% elongation). Membrane C/PC levels were measured with GS/MS and a fluorometric PC assay. Normalized relative to unstimulated controls, the stretched samples showed time-dependent decrease in membrane cholesterol content to 0.58 ± 0.089 (1 h; N = 3; *P* < 0.01) and 0.30 ± 0.02 (3 h; N = 3; *P* < 0.001) (Fig. 2A, B), whereas the PC content increased to 1.32 ± 0.02 (1 h; N = 3; *P* < 0.0001) and 1.58 ± 0.03 (3 h; N = 3; *P* < 0.01), respectively (Fig. 2C). The membrane C/PC ratio (control, C/PC = 1) decreased to 0.44 following 1 h and 0.19 after 3 h of mechanical stimulation. Its sensitivity to the mechanical strain suggests that mechanical properties of the cell membrane reflect the history of exposure to the biomechanical environment.

### Reduction in free membrane cholesterol results in lipid raft loss

Membrane cholesterol was modulated with MβCD, a water-soluble cyclic oligosaccharide that encapsulates hydrophobic membrane cholesterol residues and has been widely used to characterize cholesterol dependence of ion channels (37). Endogenous unesterified cholesterol within lipid rafts was visualized with filipin, a fluorescent polyene antibiotic (51, 52). About 60 min of incubation with MβCD (10 mM) reduced filipin-positive puncta by 75.63% ± 2.77% (*P* < 0.0001) (Fig. 3A, B, and D), with cells remaining viable and responsive to physiological stimuli throughout a typical experiment (∼1–3 h). Cholesterol:MβCD supplementation (1:10) (38) increased the number of filipin-positive puncta by 6.17 ± 0.67-fold (Fig. 3C, D). These data show that formation of raft domains in TM cells is predicated upon free membrane cholesterol levels.

### Reduction in free membrane cholesterol is associated with upregulated expression of F-actin

Mechanical stability and structural integrity of cells are maintained by cortical actin (arrowheads in Fig. 3A) and ventral stress fibers (arrows), which are often reinforced in response to mechanical stress (19, 24, 27). We tested whether altering membrane stiffness through cholesterol depletion/enrichment impacts cytoskeletal architecture in cells labeled with phalloidin-actin Alexa 488. One-hour exposure to 0.1–10 mM MβCD was associated with dose-dependent increases in stress fiber fluorescence. Exposure to 10 mM cyclodextrin increased the F-actin signal by 55.8 + 9.5% (Fig. 3B, E) (N = 4; *P* < 0.001), whereas supplementation with saturated 1:10 (1 mM) mixture of cholesterol and MβCD did not affect actin expression (Fig. 3C, E) despite the sizeable increase in the number of filipin^+^ puncta (Fig. 3C, D). Thus, actomyosin organization in TM cells does not appear to require lipid rafts.

### Cholesterol depletion facilitates agonist-induced TRPV4 activation

TRPV4, a polymodal calcium-permeable channel that recently emerged as potential regulator of TM pressure, strain and volume sensing, and conventional outflow (20, 26, 27) contains putative cholesterol recognition motifs within Loop4-TM5 (30). Its sensitivity to cholesterol modulation was investigated in cells loaded with the Ca^2+^ indicator dye Fura-2-AM and stimulated with the agonist GSK1016780A (GSK101) before and after exposure to MβCD. As previously shown (26, 27), GSK101 (25 nM) reversibly increased [Ca^2+^]_TM_ (Fig. 4A). MβCD increased the peak amplitude of the GSK101-evoked signal from 0.5 ± 0.04 (n = 39; N = 6) to 1.06 ± 0.13 (n = 53; N = 6; *P* < 0.0001) (Fig. 4A, B) (∼100% increase) without affecting baseline [Ca^2+^]_i_. Agonist stimulation under cholesterol-enriched conditions produced a small but significant increase in the average GSK101 response (0.74 ± 0.05; n = 44; N = 5; *P* < 0.001) (Fig. 4A, B).

**Supplemental Fig. S1:**
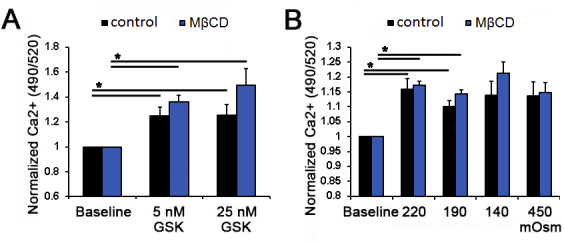
Averaged, maximum fluorescence data in Fluo-4-loaded TM cells. A: Spectrophotometry shows dose-dependent [Ca^2+^]_i_ increases in response to 5 and 25 nM GSK and augmentation by cholesterol removal (N = 4). B: Data show dose-dependent [Ca^2+^_]i_ increases in response to different HTS solutions and augmentations by cholesterol removal (N = 3). ∗*P* < 0.05. [Ca^2+^]_i_, intracellular calcium concentration;HTS, hypotonic stimuli.

Spectrophotometry was used to assess the effects of cholesterol depletion on the TM population response. About 5 nM GSK101 produced a 24.8 ± 8.31% increase in the Fluo-4 fluorescence signal (N = 4 independent experiments; *P* < 0.05; supplemental Fig. S1A). MβCD potentiated the response by ∼12%, resulting in 36.27 ± 5.18% increase compared with the baseline (*P* < 0.05) (Fig. 4C). A similar facilitatory effect was observed with 25 nM GSK101 (supplemental Fig. S1A). These data suggest that cholesterol suppresses agonist-induced TRPV4 activation.

### Cholesterol modulates swelling-induced calcium signaling

Originally identified as a regulator of cellular swelling (53, 54), TRPV4 functions as a real-time readout of cell volume changes (48) with osmoregulatory functions in neurons, glia, epithelial, and endothelial cells (40, 41, 55, 56, 57, 58). To determine the effect of raft disruption on the TM swelling response, we exposed the cells to HTS in the presence/absence of MβCD. Consistent with TRPV4 activation, TM cells responded to 140 mOsm HTS with significant (0.44 ± 0.06; n = 24; N = 4; *P* < 0.0001) increases in [Ca^2+^]_i_ (Fig. 5A, B). Depletion of cholesterol doubled the peak response amplitude to 0.87 ± 0.08 (n = 27; N = 4; *P* < 0.001), with the facilitatory effect inhibited by cholesterol supplementation (0.43 ± 0.04; n = 30; N = 4; *P* < 0.001) (Fig. 5A, B). Spectrophotometry similarly showed dose-dependent [Ca^2+^]_i_ increases in response to HTS (in case of 55% HTS: 1.14 ± 0.03; N = 3) and augmentation by cholesterol removal (to 1.21 ± 0.022; N = 3) (Fig. 5C and supplemental Fig. S1B) (*P* < 0.05).

### Cholesterol modulates the TM response to membrane strain

**Supplemental Fig. S2:**
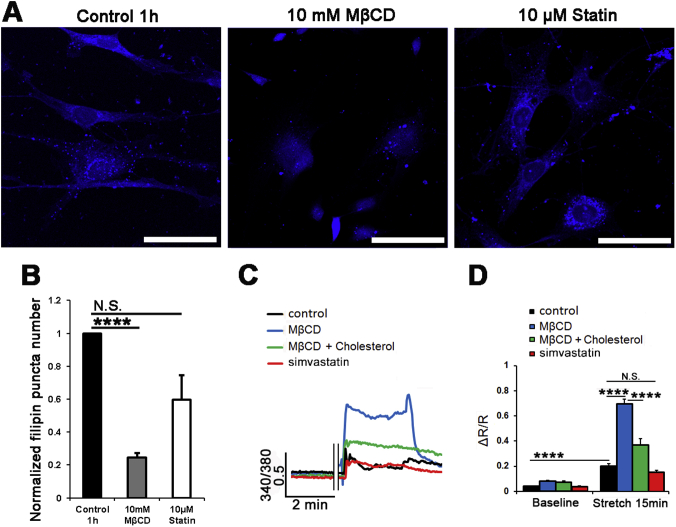
Membrane cholesterol modulates cyclic stretch–induced [Ca^2+^]_i_ response. A: Representative traces and (B) averaged results for 15-min cyclic stretch of control (black trace, bars), MβCD-treated (10 mM, blue trace, bar), MβCD:cholesterol–treated (green trace, bar), and simvastatin-treated (1 mM, red trace, bar) cells. Stretch-evoked [Ca^2+]^_i_ responses are facilitated by MβCD, whereas MβCD:cholesterol (green bar) decreased the amplitude of the response. Simvastatin (1 mM) did not facilitate the stretch effect (n = 21-77; N = 5-6); ∗∗∗∗*P* < 0.0001. The scale bar represents 20 μm. [Ca^2+^]_i_, intracellular calcium concentration; MβCD, m-β-cyclodextrin.

We next investigated whether cholesterol depletion impacts the transduction of cyclic mechanical stretch, which mirrored strains impelled on ECM beams by IOP fluctuations (59, 60, 61) that are transduced partly via TRPV4 (20, 27). Cells were stimulated with periodic and calibrated displacements/relaxations of the collagen IV-coated substrate (1 Hz; 10% elongation, 15 min). In response to stretch, cytosolic [Ca^2+^]_i_ reversibly increased to 0.21 ± 0.02 (n = 21; N = 6; *P* < 0.0001) (Fig. 1A, B). The effect was augmented by cholesterol depletion to 0.71 ± 0.04 (n = 77; N = 5; *P* < 0.0001) and reduced by cholesterol supplementation to 0.38 ± 0.05 (n = 37; N = 6; *P* < 0.0001) (Fig. 1A, B). Simvastatin (10 μM), an inhibitor of 3-hydroxy-3-methylglutaryl coenzyme A reductase, the rate-limiting endogenous enzyme in cholesterol biosynthesis, induced a small and nonsignificant decrease in the number of fillipin^+^ puncta (supplemental Fig. S2A, B). Accordingly, the statin did not affect the amplitude or kinetics of stress-induced [Ca^2+^]_i_ elevations (0.16 ± 0.01; n = 12; N = 2) (supplemental Fig. S2C, D).

**Fig. 6:**
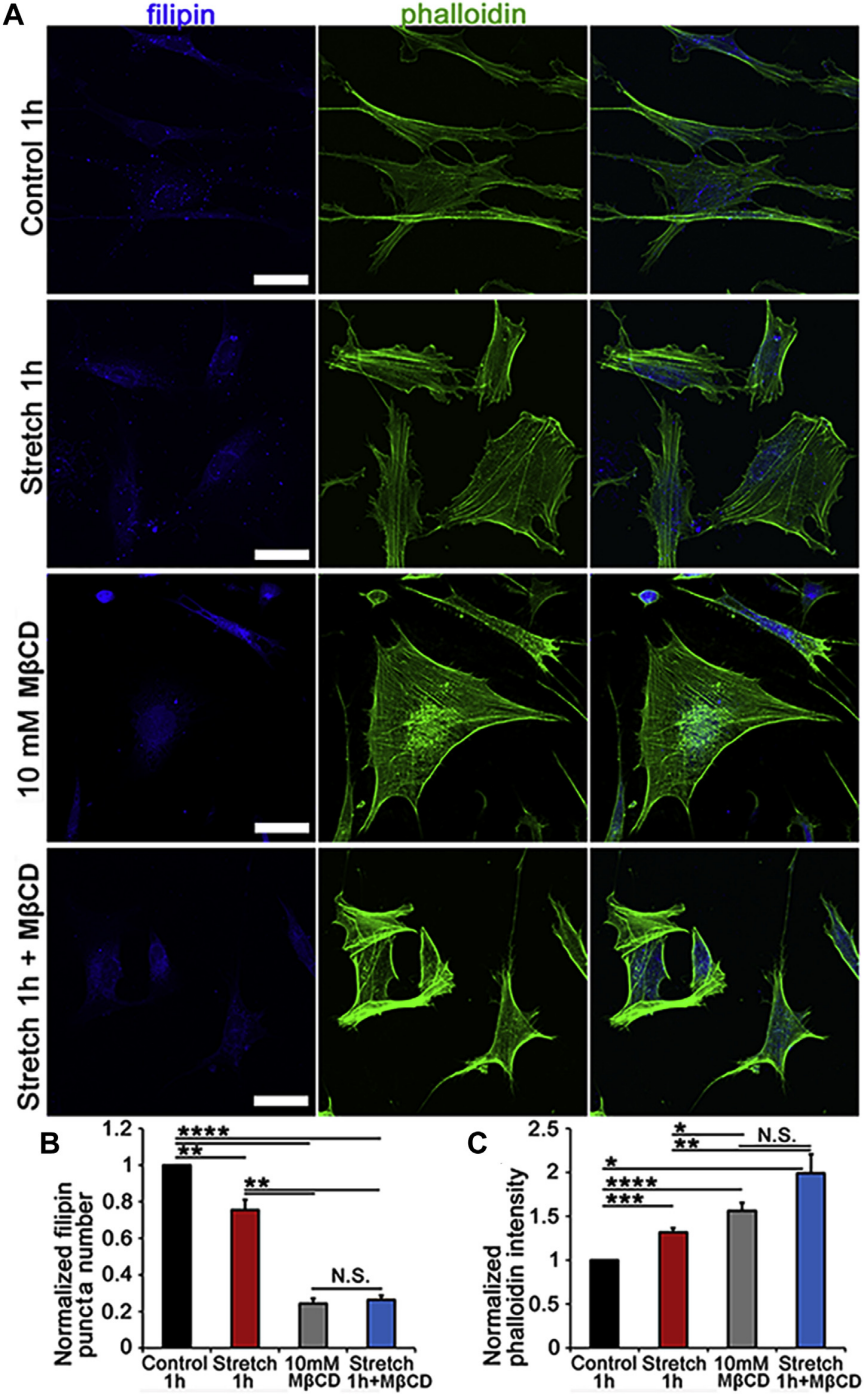
Membrane cholesterol content regulates stretch dependence of cytoskeletal remodeling. A: TM cells were stretched in the presence/absence of MβCD and MβCD:cholesterol and labeled for filipin (405 nm) and phalloidin-Alexa 488 nm in control, 6% stretch (0.5 Hz, 6%, 1 h), MβCD and MβCD+ stretch samples. B: Averaged and normalized puncta number. Filipin is significantly reduced by stretch and MβCD alone. C: Stretch and MβCD significantly facilitate F-actin fluorescence. Combined stimulation results in an additional ∼30% increase in stress fiber signal (N = 3–4). ∗*P* < 0.05; ∗∗*P* < 0.01; ∗∗∗*P* < 0.001; ∗∗∗∗*P* < 0.0001. The scale bar represents 20 μm.

Changes in membrane cholesterol content have been shown to influence cell surface tension and actomyosin assembly (62). To test the effect of membrane cholesterol on stress fibers, F-actin was labeled with fluorescent phalloidin following exposure to cyclic stretch in the presence/absence of MβCD and MβCD:cholesterol. As shown previously, stretch alone augmented phalloidin-actin fluorescence (to 1.32 ± 0.05; N = 3; *P* < 0.001) (Fig. 6A, C). Interestingly, this was associated with a significant decrease in the density of filipin puncta (to 0.76 ± 0.05; N = 3; *P* < 0.01) (Fig. 6A, B), suggesting that mechanical stress influences the formation of lipid rafts. As shown in Fig. 3, MβCD increased the F-actin signal (1.56 ± 0.09; N = 3; *P* < 0.0001) while reducing filipin fluorescence to 0.24 ± 0.03 (N = 3; *P* < 0.0001). MβCD treatment of stretch-exposed cells did not further affect filipin fluorescence (0.26 ± 0.02; N = 3) (Fig. 6A, B), whereas stress fiber fluorescence showed a significant 42.6% ± 21.6% increase over the effect of stretch alone (N = 3; *P* < 0.05) (Fig. 6C). These data suggest that stretch and cholesterol depletion facilitate F-actin expression in an additive manner and that stretch functions as a negative regulator of raft assembly.

### Membrane cholesterol regulates TRPV4 expression

**Fig. 7:**
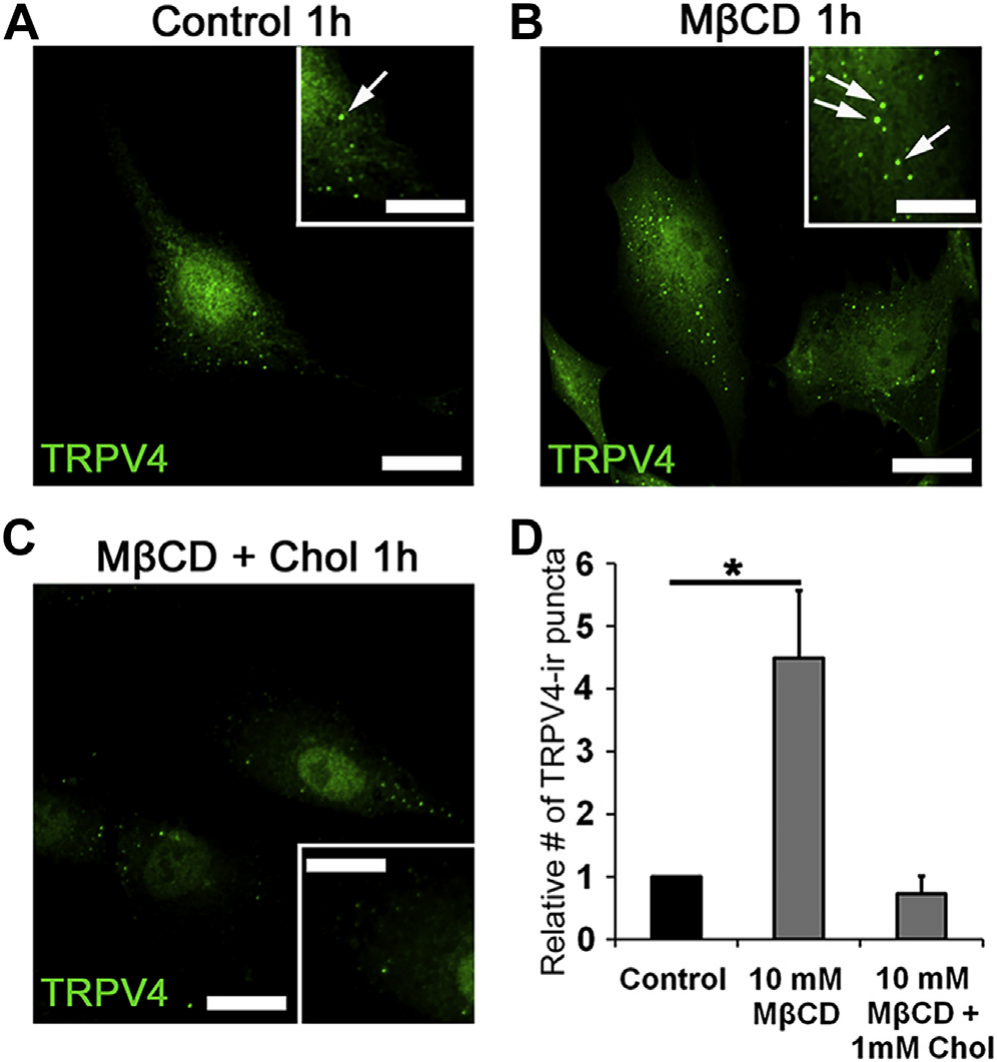
Cholesterol depletion increases the number of TRPV4-ir puncta. TRPV4 immunolabeling of primary TM cells. Representative examples of (A) control, (B) 1 h treatment with MβCD, and (C) 1 h treatment with MβCD:cholesterol. Inset: zoomed-in region with TRPV4-ir puncta (arrow). D: Summary of three independent experiments, normalized for control cells. The number of TRPV4 puncta is upregulated after incubation with MβCD. ∗*P* < 0.05. The scale bar represents 10 μm.

Depending on the cell type and protein isoform, cholesterol-modulating agents potentiate or inhibit TRP channel trafficking (63, 64, 65). We tested how free membrane cholesterol impacts TRPV4 channel expression in fixed cells labeled with a validated antibody (40, 42). One-hour incubation with MβCD produced a 4.49 ± 1.07-fold increase in the number of TRPV4-ir (immunoreactivity [ir]) puncta (N = 3; *P* < 0.05) (Fig. 7 A, B, D). Cholesterol enrichment abrogated this effect (0.73 ± 0.28; N = 3; *P* < 0.05) (Fig. 7C, D).

### MβCD potentiates TRPV4 current in heterologously expressing oocytes

The opening of the TRPV4 channel pore in mammalian cells reflects many simultaneous inputs, including mechanical stressors, temperature, polyunsaturated fatty acids, and accessory binding proteins (58, 66, 67). To isolate the effects of cholesterol on the channel from auxiliary proteins and intracellular signaling within TM cells, we determined the cholesterol dependence of TRPV4 currents in the *Xenopus laevis* expression system that has been used in previous studies of cholesterol depletion (68) and TRPV4 signaling (40, 41, 48). Because oocyte viability is compromised at millimolar MβCD concentrations (68), the experiments were conducted using 50 μM MβCD.

**Fig. 8:**
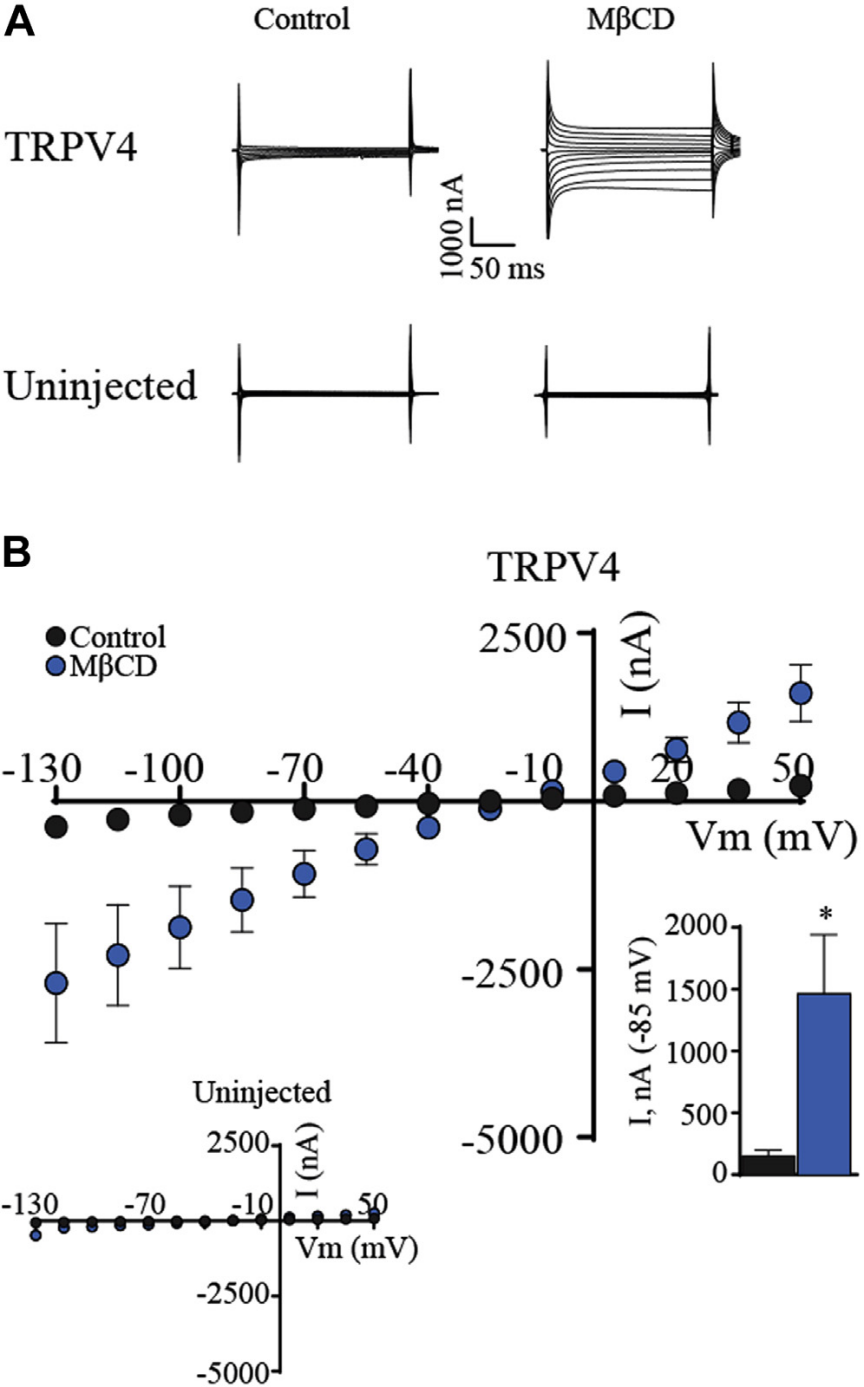
Cholesterol depletion enhances TRPV4-mediated membrane currents in *Xenopus* oocytes. A: Representative current traces from TRPV4-expressing oocytes and uninjected control oocytes in control solution or after 45 min exposure to 50 μM MβCD. B: I/V curves of TRPV4-expressing oocytes exposed to control solution or MβCD, with uninjected oocytes in inset. Summarized currents obtained at −85 mV are shown in the lower inset. The magnitude of TRPV4-mediated currents (at *V*_m_ = −85 mV) was compared using Student's *t*-test. ∗∗*P* < 0.01; N = 9. NS, not significant.

Membrane currents in uninjected control oocytes and TRPV4-expressing *Xenopus* oocytes were monitored by standard two-electrode voltage clamp. A voltage step protocol demonstrated small tonic currents in TRPV4-expressing oocytes (Fig. 8A, upper left panel) compared with those obtained in uninjected oocytes (Fig. 8A, lower left panel), summarized in Fig. 8B and inset, N = 9. Transmembrane currents of uninjected oocytes were undisturbed by MβCD exposure, whereas those of the TRPV4-expressing oocytes were enhanced approximately 10-fold (from 156 ± 42 to 1,466 ± 475 nA, N = 9, *P* = 0.014) by cholesterol depletion (Fig. 8A [right panels], B). These data indicate that membrane cholesterol suppresses tonic TRPV4 activity, with insufficiency thereof enhancing the TRPV4-mediated membrane currents.

### TRPV4 is predominantly outside the caveolae/lipid rafts

**Fig. 9:**
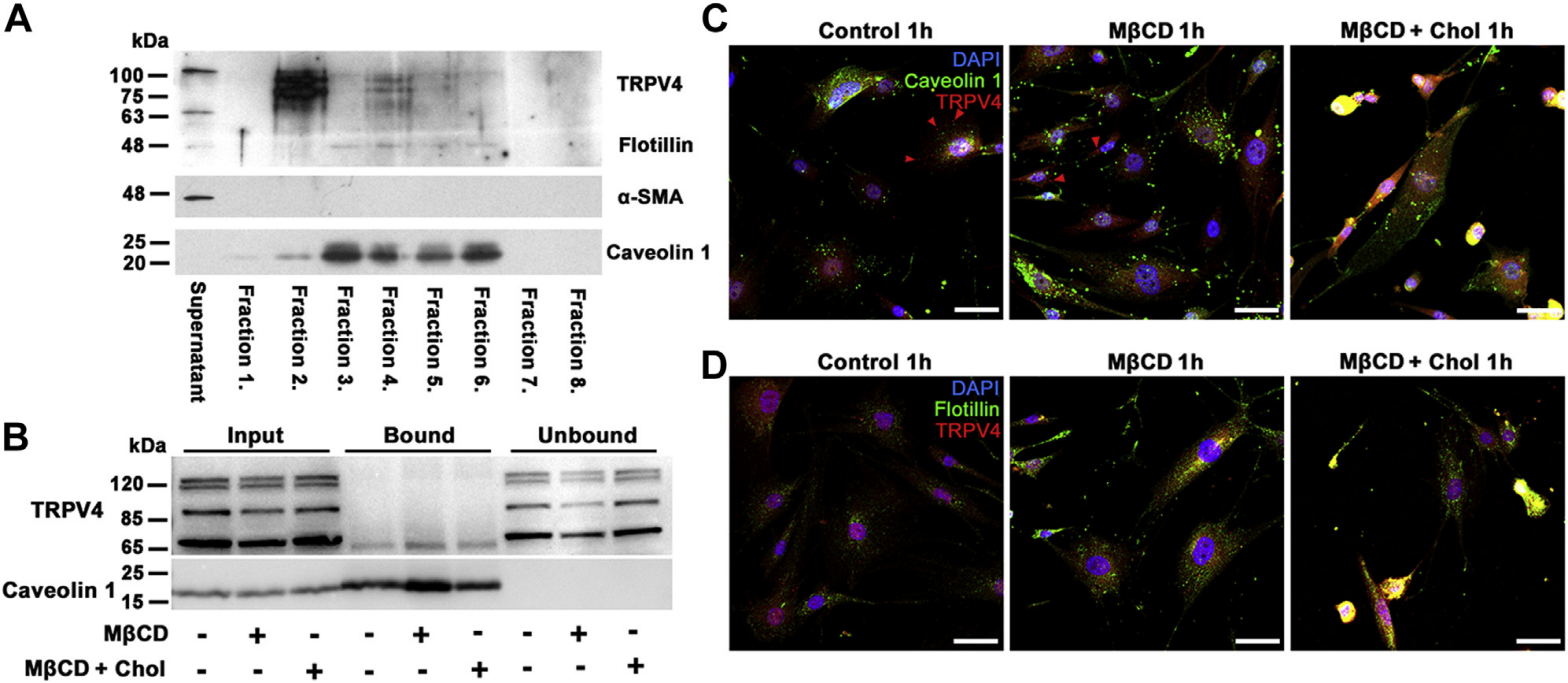
Most membrane TRPV4 does not partition into raft domains or interact with caveolar proteins. A: Western blot; detergent-free lipid raft isolation in primary TM cells. The supernatant fraction contains cytosolic proteins; fractions 1 and 2 (5% sucrose) contain nonlipid-raft membrane proteins, fractions 3–6 (5–35% sucrose) contain lipid raft membranes, and fractions 7 and 8 (pellet, 45% sucrose) contain unsuspended proteins and cell nuclei. TRPV4 protein is predominantly confined to fraction 2; fraction 4 is associated with flotillin-1 (48 kDa) and Cav-1 (22 kDa), whereas the supernatant/cytosol fraction associates with α-SMA (42 kDa). B: Coimmunoprecipitation. TRPV4-Cav-1 interaction assessed with the Cav-1 antibody for TRPV4 pulldown in control, MβCD, and MβCD:cholesterol-treated samples. Input bands, whole-cell lysate; bound bands, Cav-1-bound protein fraction; unbound bands, flow-through fractions. Cav-1-bound fractions show modest precipitation of the ∼75 kDa TRPV4 isoform. The absence of Cav-1 expression in unbound fractions confirms the quantitative immunoprecipitation of Cav-1. C, D: Immunohistochemistry, for control, MβCD, and MβCD:cholesterol-treated cells. C: Double immunolabeling for TRPV4 and Cav-1. TRPV4-ir (red arrowheads) does not colocalize with Cav1-ir puncta (green). D: Double immunolabeling for TRPV4 and flotillin. TRPV4-ir (red puncta) does not colocalize with flotillin-ir puncta (green). The inset is shown at higher magnification insets as supplemental Fig. S3. The scale bar represents 20 μm.

To determine whether TRPV4 is enriched in lipid rafts, detergent-free lipids were isolated by gradient (5%/35%/45% sucrose) ultracentrifugation, and fractions were analyzed by Western blot. Flotillin-1, Cav-1, and α-SMA antibodies were used as markers for noncaveolar lipid, caveolar lipid, and cytosolic fractions, respectively. The 48 kDa noncaveolar lipid raft marker flotillin-1 labeled fractions 3–6. The caveolar marker Cav-1 (∼22 kDa) predominantly labeled fractions 3–6, with weak signal present in fractions 1 and 2, whereas α-SMA (∼42 kDa) was confined to the supernatant/cytosol fraction. The TRPV4 protein mainly partitioned into the flotillin-free fraction 2, which showed a weak Cav-1 signal (Fig. 9A). Thus, by far the main fraction of membrane TRPV4 is located in nonraft domains that lack caveolar and noncaveolar markers. It is possible that a limited amount of Cav-1 protein shares the membrane with TRPV4 in fraction 2 (Fig. 9A), without direct interaction between the proteins (Fig. 9B, C).

TRPV4 in cultured endothelial cells coimmunoprecipitates with Cav-1 (31, 32). To test the interaction in TM cells, Cav-1 was immunoprecipitated from TM cell lysates, and association with TRPV4 was assessed by Western blotting. The four bands in the Western blot (Fig. 9B) presumably correspond to glycosylated, full-length, and truncated protein (TRPV4A-E, (69, 70, 71)). Following quantitative precipitation of Cav-1, a portion of the ∼75 kDa truncated variant associated with Cav-1, whereas all the full-length variants observed in the total lysate partitioned to Cav-1-depleted unbound fractions (Fig. 9D). Thus, the most significant fraction of TRPV4 by far localizes to noncaveolar nonraft membrane domains.

Raft/caveolar localization of TRPV4 was examined further with immunochemistry. TRPV4-ir did not colocalize with Cav-1 (Fig. 9C, red arrowheads) and flotillin (Fig. 9D), thereby confirming that TRPV4 does not localize into the raft domains. Cholesterol depletion increased the number of Cav-ir puncta without promoting caveolar TRPV4 translocation.

## Discussion

In this study, we demonstrate that the mechanical milieu regulates the lipid composition of the membrane and that TRPV4, a nonselective cation channel that mediates a wide range of physical and chemical inputs, is highly sensitive to free membrane cholesterol levels. The key findings are *i*) physiological levels of mechanical stretch regulate the TM membrane C/PC ratio, *ii*) the majority of TRPV4 protein is excluded from raft/caveolar regions, with the possible exception of a small proportion of a truncated splice variant that interacts with Cav-1; *iii*) membrane cholesterol negatively modulates TRPV4 activation by agonists and mechanical stimuli; *iv*) TM F-actin expression is cholesterol and stretch dependent; and *v*) cholesterol tonically suppresses TRPV4 activity in a nonmammalian expression system. These findings implicate cholesterol-TRPV4 interactions in dynamic and use-dependent regulation of cellular mechanosensing.

In contrast to the extensive body of knowledge about how lipid composition affects the structural and mechanical properties of biological membranes, much less is known about the relationship between mechanical stress and membrane lipid content. Our finding that cyclic strain lowers the membrane C/PC ratio suggests that biophysical and modulatory properties of biological membranes reflect the biomechanical environment. The sensitivity of stretch-induced reorganization of the lipid content to HC067047 further identifies TRPV4 activation as an obligatory step that couples mechanical strain to lipid signaling. Our second conclusion is that changes in the lipid content may profoundly modulate function of embedded mechanoregulated proteins such as TRPV4. This could be relevant in blinding diseases such as glaucoma, which has been associated with elevated C/PC ratios in TM cells (22).

Free cholesterol is a central constituent of caveolae and lipid rafts, which serve as regulatory portals for cellular cholesterol homeostasis (1, 2, 5, 7, 72). Its depletion results in near-total dissolution of lipid rafts (visualized by filipin fluorescence) and profoundly modulates Ca^2+^ signals induced by the TRPV4 agonist GSK1016790A, cyclic strain, and cell swelling. Given that MβCD similarly facilitates transmembrane currents in TRPV4-expressing, but not control, *X. laevis* oocytes, we propose that the channel is directly and negatively regulated by membrane cholesterol. Supplementation with exogenous MβCD:cholesterol obviated the facilitation induced by the cyclodextrin alone without affecting peak amplitudes of agonist-evoked and mechanically evoked Ca^2+^ signals. This may reflect saturation of control membranes with free cholesterol, which constitutes approximately a third of the total membrane lipid mass (5, 38). A similar conclusion was reached for the modulation of the Piezo1 channel, which is modulated by MβCD but not MβCD:cholesterol (73). The ∼4.5-fold increase in the number and intensity of TRPV4-ir puncta (Fig. 7) suggests that cholesterol regulates transport and/or retrieval of TRPV4-containing vesicle pools, as previously shown for TRPV1 (65, 74), Kv1.5 (75), Kir (39), LRRC8/SWELL (76), and TRPC3 (77) channels. Another possibility is that cholesterol regulates formation of clathrin-coated vesicles (78) and endocytosis/secretion via PACSIN, which binds the N terminus of TRPV4 (75).

Lipids regulate the gating of ion channels through direct interactions within membrane microdomains and indirectly by shaping the biophysical properties of the membrane. An increase in membrane tension, caused by cholesterol removal, might facilitate opening of stretch-activated channels (79, 80) yet viscoelastic “force-from-lipid” models (77, 81, 82) cannot explain why ion channels respond differently to cholesterol enantiomers with similar effects on membrane properties (83). In contrast to its facilitation of TRPV4 signals in TM cells and oocytes, cholesterol depletion *suppresses* TRPV4 activation in glia (45) and endothelial cells (31). Consistent with allosteric modulation of channel subunits, accessory proteins, and/or residues buried within the bilayer, MβCD facilitates activation of mammalian TRPM8 (63), TRPM3 (64), TRPC3 (77), and volume-activated chloride (76) channels but inhibits TRPA1, TRPC1, TRPC6 (84, 85, 86), and blocks TRPL channels in fly photoreceptors (87). Allosteric sites may include cholesterol recognition/interaction amino acid consensus-like (KDLFRFLL) recognition motifs that span loop 4-TM5 of TRPV4 (30, 88, 89). Consistent with our results, loss of TRPV4-cholesterol interaction in the TRPV4^R616Q^ inverted cholesterol recognition/interaction amino acid consensus motif mutation was associated with gain of function for TRPV4 (90). Cholesterol thus appears to regulate the context of the cellular sensory response such that TRPV4 activity in yeast (which cannot synthesize cholesterol) responds to swelling but not temperature (91), whereas the mammalian channel is optimally active at ∼34–28°C. Similar context dependence was observed in the TRPV1 channel, in which MβCD inhibits capsaicin-evoked and proton-evoked currents (92, 93) while facilitating its sensitivity to thermal inputs (92).

Mechanosensing often involves interactions between the cell membrane and the cytoskeleton. Our finding that MβCD treatment dissolves lipid rafts and stimulates actin polymerization in TM cells accords with its effects on osteoblasts, fibroblasts, endothelial cells, and myocytes (62, 94, 95). The additivity of the effects of stretch and cholesterol depletion (Fig. 6C) suggests that stretch-activated channels and cholesterol-regulated membrane domains signal via independent mechanisms that converge at the cytoskeleton in order to dynamically regulate membrane/cell stiffness (25, 26, 27, 96). In contrast to MβCD, simvastatin (which blocks the synthesis of the cholesterol precursor mevalonate) did not affect the amplitude of stretch-induced Ca^2+^ signals. Differential effects of simvastatin versus MβCD on stress fiber formation and cell contractility have been reported in endothelial cells and fibroblasts (94, 97).

The outflow of aqueous humor is fine tuned by arrays of mechanosensitive molecules that include lipid rafts, caveolae, focal complexes, changes in gene expression, ECM and TRPV4, Piezo1, and TREK-1 channels (26, 33, 98, 99, 100, 101, 102, 103). TRPV4 in endothelial and smooth muscle cells was reported to interact with Cav-1 (31, 32, 90), and both proteins regulate TM mechanosensitivity and outflow homeostasis (26, 27, 104, 105, 106). However, our hypothesis that TM cells manifest TRPV4-Cav-1 interactions in cholesterol-enriched raft/caveolar domains was not confirmed. We found that TRPV4 does not colocalize with filipin (lipid raft marker), Cav-1 (a marker of caveolae), flotillin (marker of noncaveolar rafts), or αSMA (cytosolic protein marker), and that the majority of the TRPV4 protein, which consists of 871 amino acids with at least five variants with unknown differences in function (70, 71, 107), partitions into fractions that exclude raft proteins. Glycosylated full-length variants (69, 70, 71) constitute by far the most significant fraction of the protein and partition into total lysate/unbound fractions that do not contain Cav-1/flotillin and resist cholesterol depletion (Fig. 9D). Interestingly, Cav-1 did precipitate a small portion of the ∼75 kDa variant, suggesting that TRPV4 variants might be differentially susceptible to caveolar interaction. Our findings suggest that loss of cholesterol interaction in nonraft regions results in a gain of function for TRPV4 activation (90), as previously shown for the TRPM8 channel, which shows enhanced gating in nonraft domains (63). It remains to be seen whether TRPV4 variants differ in microdomain interactions with lipids, G-proteins, protein kinases/phosphatases, and/or Ca^2+^-binding proteins.

In summary, our study suggests that TM mechanotransduction is integrated with cholesterol homeostasis. The interdependence between membrane tension, cholesterol content, TRPV4 signaling, and calcium homeostasis suggests that mechanical sensing is a highly dynamic and integrated process that could influence regulation of the conventional outflow pathway. Cholesterol may protect the TM from hypertension-induced injury by downregulating the sensitivity to mechanical stress, but this adaptive function could be compromised by cholesterol dysregulation in glaucoma, diabetic retinopathy, Niemann-Pick disease, and/or macular degeneration.

## Data availability

The data described are contained within the article.

## Supplemental data

This article contains supplemental data.

## Conflict of interest

The authors declare that they have no conflicts of interest with the contents of this article.

## Figures and Tables

**Supplemental Fig. S3:**
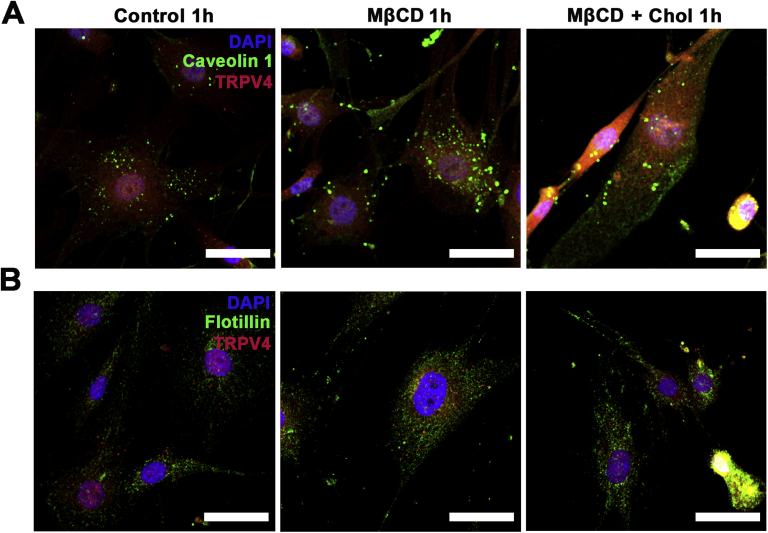
Most membrane TRPV4 does not partition into raft domains or interact with caveolar proteins. Higher magnification insets of images Fig. 9C, D. (*A*) double immunolabeling for TRPV4 and Cav-1; cell nuclei are labeled with DAPI. (*B*) double immunolabeling for TRPV4 and flotillin; cell nuclei are labeled by DAPI. The scale bar represents 20 μm. DAPI, 4',6-diamidino-2-phenylindole; MβCD, m-β-cyclodextrin; TRPV4, transient receptor potential vanilloid isoform 4.
